# Journal Impact Factor: Do the Numerator and Denominator Need Correction?

**DOI:** 10.1371/journal.pone.0151414

**Published:** 2016-03-15

**Authors:** Xue-Li Liu, Shuang-Shuang Gai, Jing Zhou

**Affiliations:** 1 Henan Research Center for Science Journals, Xinxiang Medical University, Xinxiang, Henan Province, China; 2 Periodicals Publishing House, Xinxiang Medical University, Xinxiang, Henan Province, China; 3 Management Institute, Xinxiang Medical University, Xinxiang, Henan Province, China; Universidad de Las Palmas de Gran Canaria, SPAIN

## Abstract

To correct the incongruence of document types between the numerator and denominator in the traditional impact factor (IF), we make a corresponding adjustment to its formula and present five corrective IFs: IF_Total/Total_, IF_Total/AREL_, IF_AR/AR_, IF_AREL/AR_, and IF_AREL/AREL_. Based on a survey of researchers in the fields of ophthalmology and mathematics, we obtained the real impact ranking of sample journals in the minds of peer experts. The correlations between various IFs and questionnaire score were analyzed to verify their journal evaluation effects. The results show that it is scientific and reasonable to use five corrective IFs for journal evaluation for both ophthalmology and mathematics. For ophthalmology, the journal evaluation effects of the five corrective IFs are superior than those of traditional IF: the corrective effect of IF_AR/AR_ is the best, IF_AREL/AR_ is better than IF_Total/Total_, followed by IF_Total/AREL_, and IF_AREL/AREL_. For mathematics, the journal evaluation effect of traditional IF is superior than those of the five corrective IFs: the corrective effect of IF_Total/Total_ is best, IF_AREL/AR_ is better than IF_Total/AREL_ and IF_AREL/AREL_, and the corrective effect of IF_AR/AR_ is the worst. In conclusion, not all disciplinary journal IF need correction. The results in the current paper show that to correct the IF of ophthalmologic journals may be valuable, but it seems to be meaningless for mathematic journals.

## Introduction

One of the most famous researchers in bibliometrics, Garfield [1] first proposed the term of impact factor (IF) in his paper published in *Science* in 1955. At first, IF meant citations to articles, not the current IF used for evaluating journals [2]. Journal IF is the natural result of the establishment of the Science Citation Index (SCI) database. In the 1960s, supported by the US National Institutes of Health, the Genetics Citation Index was established successfully, which led to the direct generation of the SCI database. Garfield and Sher then proposed IF as an indicator for evaluating the academic impact of journals in 1963, and IF was applied to assist the selection of source journals in the SCI database [3–5]. In 1972, Garfield [6] formally established the concept and formula of journal IF. However, IF really became a journal evaluation indicator in 1975, when the Journal Citation Reports (JCR) was established, and ever since has been one of the most authoritative scientometric indicators for assessing the international status and academic impact of journals [7].

To be exact, IF is defined as the number of citations within a given year of items published by a journal in the preceding two years divided by the number of citable items published by the journal in these two years [8]. Obviously, the numerator of IF is the number of citations of all types of items, whereas the denominator just includes citable items. Thus, it is undeniable that the design formula of IF is not perfect, and this fact has attracted much attention and aroused much controversy in academia.

Thomson Reuters divides source items into citable items and non-citable items. Citable items include only original research articles and reviews. Meeting abstracts, editorials, letters, news items, corrections, book reviews, biographical items, reprints, and so on are all rated as non-citable items [9–11]. In fact, non-citable items are not uncitable, some that are published by many journals, especially several prestigious international journals, are cited frequently and do contribute to a journal’s citations [12–13]. When these non-citable items are cited, their citations are counted in the numerator of IF, but are excluded in the denominator. Therefore, citations to non-citable items are usually known as a “free lunch” [14–17].

Many scholars have noticed this defect of the IF and questioned its fairness and reasonability for journal evaluation. Bensman [18] indicated that distinguishing citable and non-citable items was a fatal flaw that has existed since IF was first presented. Some scholars like Campanario [19–21] believe IF is a biased indicator and have pointed out that non-citable items have an important influence on it, and as a consequence the IF of a considerable number of journals including some of the most prestigious ones might be inflated by 30%–40%. Some researchers have pointed out that the incongruence of source items between the numerator and denominator seriously distort the true IF value of journals, and cause its manipulation and abuse [22–23]. More incredibly, one has pointed out that the number of the citable items in the denominator could be negotiated with Thomson Reuter, which might result in an IF variation of more than 300% [24–25].

Some scholars have presented several suggestions and solutions based on the inconsistency of source items in IF, and these fall into three main approaches, as follows:

1. Releasing new journal evaluation indicators. The Scopus database launched the SJR and SNIP indicators in 2007 and 2010. Similar to IF, these two new indicators are average citations to journals. However, they break some of the limitations of IF, such as prolonging the citation time window to three years and considering the citation peaks of different subjects. Among them, one of the most obvious advantages is their consistency in the source items between numerator and denominator, which counts reviews and articles, including proceedings papers [26–27]. Based on the principle and algorithm of weights based on order relation and experts’ suggestions, Xu and Fang [28] constructed a weighted IF that changed the order relation of different document types, including non-citable items.
2. Computing the IF per type of document. Moed et al. [29] found that the IF of *The Lancet* was reduced by almost 40% when just counting citations of the citable items, and *Nature*’ IF would be reduced by some 30% by including letters. Hence, they concluded that IF depended strongly on the set of documents used and suggested having a separate calculation for the IF of each type of document.
3. Redefining the types of source items in the numerator and denominator of the IF. Wu [30] asserted that the denominator should count the quantity of all source items now that the non-citable items were citable. He proposed that the definition of IF would be better readjusted. Some scholars [31–32] have argued that the type of source items between numerator and denominator should be consistent and suggested counting citations to articles and reviews in the numerator only. Some have suggested redefining citable items such as articles, notes, letters, and reviews and just count citations to these four source items [33]. Lozano et al. [34] corrected the inconsistency of document types between the numerator and denominator when analyzing the correlation between IF and citations in the digital age.

Even so, the current studies on the inconsistency between the numerator and denominator of the IF mainly focus on academic criticisms, proposals for modifications, and selecting sample journals to compute the corrected IF and compare it with traditional IF. These studies mainly remain at theory stage, and there is no empirical research on the journal evaluation effect of corrective IFs.

In the current study, based on the quantitative and cited characteristics of non-citable items, we have selected ophthalmologic journals and mathematical journals as research objects to correct the design formula of traditional IF. Considering the fact that scholars in a country might be not familiar with some journals from other countries, we have investigated only American researchers in the fields of ophthalmology and mathematics to obtain the real impact ranking of American ophthalmologic and mathematical journals in the minds of peer experts as the “gold standard” of journal impact. The correlations between the corrective IFs and questionnaire score were analyzed to verify the corrective effect of IF in different subjects to help us determine if there is a need to correct the numerator and denominator of traditional IF. These results have important theoretical significance and practical worth to the whole research community.

## Research Methods

### Determining research objects

There are significant differences in citation behavior between different subjects. For example, mathematics usually has a later citation peak and is known as a slow moving discipline, but papers in some disciplines in medicine often reach their citation peaks quickly [35–37]. Considering that research classification and discipline boundary in ophthalmology are relatively definite and clear, we selected ophthalmologic and mathematical journals as our research objects. As mentioned before, scholars in a country might be not familiar with some journals from other countries, considering America had a large number of journals included in the JCR database in 2014, we meant to investigate American researchers in the fields of ophthalmology and mathematics to obtain the real impact ranking of American ophthalmologic and mathematical journals in the minds of peer experts. There were 30 ophthalmologic journals and 82 mathematical journals published in America. Considering a large sample size may influence the questionnaire results, we just selected comprehensive mathematical journals for our study, 27 journals.

### Obtaining the quantity and citations of different types of document

We retrieved all types of document published by each ophthalmologic and mathematical journal from 2012 to 2013, obtained the quantity per type of document through the “Refine” function in the SCI database, and obtained their citations in 2014 using the “Create Citation Report” function.

### Corrective methods of traditional IF

Based on the quantitative and citation characteristics of different type of documents in the SCI database, we propose five corrective definitions for IF as follows:

$$IF_{Total/Total}=\frac{C_{t-1}+C_{t-2}}{N_{t-1}+N_{t-2}}$$ (1)

$$IF_{Total/AREL}=\frac{C_{t-1}+C_{t-2}}{N_{AREL(t-1)}+N_{AREL(t-2)}}$$ (2)

$$IF_{AR/AR}=\frac{C_{AR(t-1)}+C_{AR(t-2)}}{N_{AR(t-1)}+N_{AR(t-2)}}$$ (3)

$$IF_{AREL/AR}=\frac{C_{AREL(t-1)}+C_{AREL(t-2)}}{N_{AR(t-1)}+N_{AR(t-2)}}$$ (4)

$$IF_{AREL/AREL}=\frac{C_{AREL(t-1)}+C_{AREL(t-2)}}{N_{AREL(t-1)}+N_{AREL(t-2)}}$$ (5)

In formulae (1) and (2), *C*_*t-1*_ and *C*_*t-2*_ are citations given in year t to all source items published in years (t–1) and (t–2), respectively, *N*_*t-1*_ and *N*_*t-2*_ are the numbers of all source items published in years (t–1) and (t–2), respectively, *N*_*AREL(t-1)*_ and *N*_*AREL(t-2)*_ are the numbers of items including articles, reviews, editorials, and letters published in years (t–1) and (t–2), respectively. In formula (3), *C*_*AR(t-1)*_ and *C*_*AR(t-2)*_ are citations in year t given to articles and reviews published in years (t–1) and (t–2), respectively, *N*_*AR(t-1)*_ and *N*_*AR(t-2)*_ are the numbers of articles and reviews published in years (t–1) and (t–2), respectively. In formulae (4) and (5), *C*_*AREL(t-1)*_ and *C*_*AREL(t-2)*_ are citations in year t given to items including articles, reviews, editorials, and letters published in years (t–1) and (t–2), respectively, and the variables *N*_*AR(t-1)*_, *N*_*AR(t-2)*_, *N*_*AREL(t-1)*_, and *N*_*AREL(t-2)*_ are defined similarly to those in formulae (2) and (3).

### Questionnaire survey

First, we selected American researchers as corresponding authors who have published ophthalmologic papers in the last 10 years or mathematical papers in the last five years in the SCI database as our respondents, and we obtained their email addresses by the field of ‘E-mail Addresses’ provided by WoS database. We obtained 9145 email addresses of ophthalmologic researchers and 7810 email addresses of mathematical researchers. We then designed a questionnaire survey about journal academic impact through the AskForm platform. Finally, we sent emails to those researchers, explained our research object, and requested them to answer the questionnaire via the provided web link. We sent successfully 7077 e-mails to American ophthalmologic researchers and 5136 e-mails to American mathematical researchers. In the questionnaire, researchers gave a score from 1.0–10.0 (the score is accurate to 1 decimal place) to each journal according to its academic impact. A score of 10 is the highest and 1 is the lowest. We calculated the total score of each journal as its real impact standard. We received 124 responses for the ophthalmologic journals and 123 responses for the mathematical journals. We excluded the questionnaires that scored less than three journals, gave the same score to all journals, or gave a score to each journal according to the sequence in which they were presented. Finally, we obtained 112 valid questionnaires for the ophthalmologic journals and 117 valid questionnaires for mathematical journals. The survey was conducted from August 4, 2015 to September 15, 2015.

### Statistical analysis method

SPSS 22.0 was used to analyze the relationships between the corrective IFs and questionnaire score using the Spearman rank correlation test.

## Results and Discussion

### Ophthalmologic Journal Results

#### Quantity and citations per type of document published by 30 ophthalmologic journals

There are significant differences in the quantity of and number of citations to each type of document for different journals. The quantity and number of citations of each type of document published by 30 ophthalmologic journals from 2012 to 2013 are shown in Table 1.

**Table 1:** Quantity and citations of each type of document published by 30 ophthalmologic journals.

Journal title	Articles		Reviews		Editorialmaterials		Letters		Other items*	
	Amount	Citation	Amount	Citation	Amount	Citation	Amount	Citation	Amount	Citation
*Invest Ophth Vis Sci*	1970	6504	9	43	55	14	84	36	35	0
*Ophthalmology*	710	4049	8	47	69	71	338	80	15	4
*Cornea*	607	1141	10	35	5	7	70	10	3	0
*Am J Ophthalmol*	555	1957	2	9	32	45	189	28	4	1
*Mol Vis*	554	1062	10	31	0	0	0	0	0	0
*J Vision*	549	696	1	8	2	0	1	0	30	4
*J Cataract Refr Surg*	545	1408	16	91	35	16	225	45	6	1
*Graef Arch Clin Exp*	541	948	11	25	9	4	147	40	5	3
*Retina-J Ret Vit Dis*	490	1365	12	73	102	73	79	4	4	0
*Optometry Vision Sci*	399	588	14	34	44	1	9	0	5	0
*Exp Eye Res*	391	987	22	94	28	16	9	7	3	0
*Ophthal Plast Recons*	323	236	5	16	22	11	95	11	0	0
*J Aapos*	286	261	2	7	14	8	39	6	9	0
*J Glaucoma*	244	384	2	6	2	3	20	1	5	0
*J Refract Surg*	234	736	9	49	13	8	49	21	9	0
*J Ocul Pharmacol Th*	213	282	17	46	14	1	9	1	0	0
*Jama Ophthalmol*	167	441	6	14	89	36	87	62	15	0
*Eye Contact Lens*	125	144	21	74	13	4	1	0	3	0
*Cutan Ocul Toxicol*	118	93	13	21	5	1	1	0	2	0
*J Neuro-Ophthalmol*	110	163	9	32	50	22	40	6	6	0
*J Ophthalmol*	108	75	45	87	7	0	0	0	0	0
*J Pediat Ophth Strab*	86	51	8	13	30	0	10	1	0	0
*Semin Ophthalmol*	84	44	40	25	1	0	1	0	0	0
*Ophthalmic Genet*	83	92	5	12	0	0	6	1	1	0
*Ocul Surf*	41	115	0	0	24	6	0	0	2	0
*Visual Neurosci*	41	57	17	53	4	1	0	0	1	0
*Optometry*	33	8	3	11	14	0	2	0	0	0
*Osli Retina*	8	9	1	0	2	0	0	0	0	0
*Surv Ophthalmol*	1	0	85	322	13	3	10	0	0	0
*Curr Opin Ophthalmol*	0	0	156	400	10	1	0	0	0	0
All journals	9616	23896	559	1678	708	352	1521	360	163	13

Note:

*means non-citable items such as biographical items, corrections and news items et al.

Overall, these ophthalmologic journals mainly published articles, followed by letters, editorials, and reviews. Articles received the highest number of citations, followed by reviews, letters, and editorials. Although the total number of letters is far higher than that of the editorials, the number of citations to both are nearly identical. Therefore, for ophthalmologic journals, editorials are more often cited than letters. As non-citable items, some editorials and letters can be cited, but biographical items, corrections, and news items are hardly ever cited. *Surv Ophthalmol* and *Curr Opin Ophthalmol* published many reviews and few editorials and letters, *Surv Ophthalmol* only published one article, and *Curr Opin Ophthalmol* did not publish any articles.

#### Ophthalmologic journal questionnaire scores and corrective IFs

We analyzed 112 valid questionnaires and obtained total expert score for the ophthalmologic journals. In addition, we calculated the five corrective IF values for each journal and retrieved its traditional IF through the JCR database. The results can be seen in Table 2.

**Table 2:** Questionnaire score and various IFs of 30 ophthalmologic journals.

Journal title	Questionnaire score	IF_Total/Total_	IF_Total/AREL_	IF_AR/AR_	IF_AREL/AR_	IF_AREL/AREL_	IF
*Invest Ophth Vis Sci*	825.4	3.064	3.115	3.308	3.334	3.115	3.404
*Am J Ophthalmol*	740.7	2.609	2.622	3.530	3.661	2.621	3.871
*Ophthalmology*	723	3.729	3.779	5.705	5.915	3.775	6.135
*Jama Ophthalmol*	636.2	1.519	1.585	2.630	3.197	1.585	3.318
*Exp Eye Res*	517	2.437	2.453	2.617	2.673	2.453	2.709
*Surv Ophthalmol*	476.6	2.982	2.982	3.744	3.779	2.982	3.849
*Graef Arch Clin Exp*	456.3	1.431	1.441	1.763	1.842	1.436	1.908
*Cornea*	431.4	1.717	1.724	1.906	1.934	1.724	2.042
*Retina-J Ret Vit Dis*	421.3	2.205	2.218	2.865	3.018	2.218	3.243
*Curr Opin Ophthalmol*	418.2	2.416	2.416	2.564	2.571	2.416	2.5
*J Cataract Refr Surg*	410.2	1.888	1.901	2.672	2.781	1.900	2.722
*J Glaucoma*	350.8	1.443	1.470	1.585	1.602	1.470	2.106
*Mol Vis*	350.8	1.938	1.938	1.938	1.938	1.938	1.986
*J Neuro-Ophthalmol*	325.5	1.037	1.067	1.639	1.874	1.067	1.95
*J Vision*	316.5	1.214	1.280	1.280	1.280	1.273	2.393
*J Ophthalmol*	314.7	1.013	1.013	1.059	1.059	1.013	1.425
*Visual Neurosci*	292.4	1.762	1.790	1.897	1.914	1.790	2.207
*J Aapos*	275.6	0.806	0.827	0.931	0.979	0.827	1.003
*J Pediat Ophth Strab*	266	0.485	0.485	0.681	0.691	0.485	0.745
*Optometry Vision Sci*	251.6	1.323	1.337	1.506	1.508	1.337	1.603
*J Ocul Pharmacol Th*	248.5	1.304	1.304	1.426	1.435	1.304	1.47
*J Refract Surg*	237.4	2.592	2.669	3.230	3.350	2.669	3.468
*Semin Ophthalmol*	211	0.548	0.548	0.556	0.556	0.548	0.863
*Ophthal Plast Recons*	210.7	0.616	0.616	0.768	0.835	0.616	0.881
*Osli Retina*	207.6	0.818	0.818	1.000	1.000	0.818	1.057
*Ocul Surf*	194.8	1.806	1.862	2.805	2.951	1.862	3.341
*Ophthalmic Genet*	190.9	1.105	1.117	1.182	1.193	1.117	1.455
*Optometry*	189	0.365	0.365	0.528	0.528	0.365	0.833
*Eye Contact Lens*	171.1	1.362	1.388	1.493	1.521	1.388	1.466
*Cutan Ocul Toxicol*	137.5	0.827	0.839	0.870	0.878	0.839	1.122

#### Correlations between the questionnaire scores and various IFs of 30 ophthalmologic journals

The evaluation of an expert in the field is considered to be the most important criterion for verifying the validity of citation indicators, and the true impact of different journals in researchers’ true estimation can be reflected by the expert scores in the questionnaire surveys [38, 39]. Hence, we can verify the effect of journal evaluation for each corrective IF by analyzing the correlations between them and the corresponding expert score.

**Fig 1:**
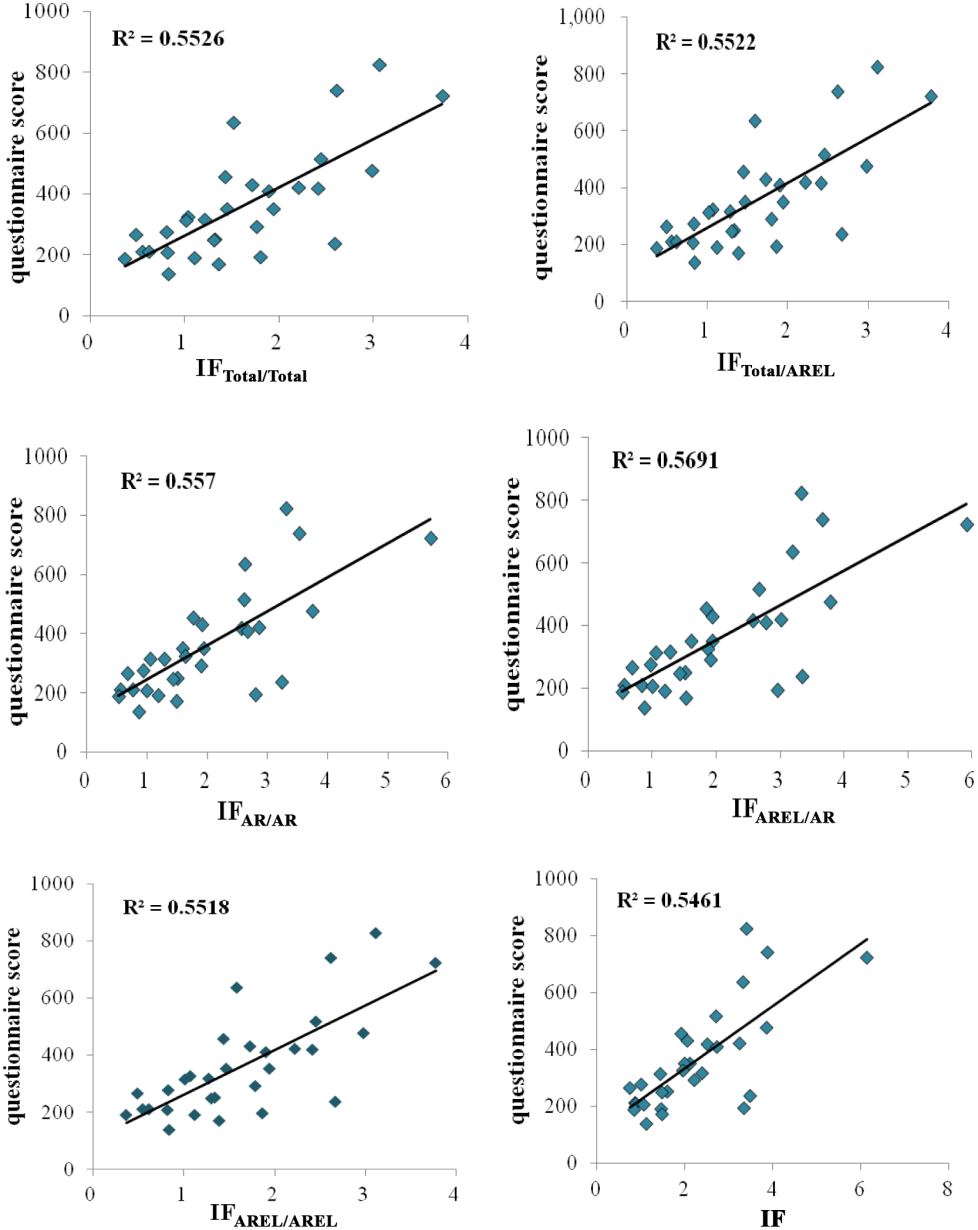
Correlations between the questionnaire score and various IFs of 30 ophthalmologic journals.

First, we illustrate whether it is reasonable to use corrective IFs for journal valuation by looking at the correlations between questionnaire score and the five corrective IFs defined above. The scatter diagrams of these relationships are presented in Fig 1. In addition, the K-S normal distribution test was conducted using the variables, and the results show that IF_AREL/AR_ is not subject to a normal distribution. Thus, we analyzed the relationships between the questionnaire score and various IFs using the Spearman rank correlation test. The results are shown in Table 3.

**Table 3:** Correlations between the questionnaire score and various IFs of 30 phthalmologic journals.

		Questionnaire score	IF_Total/Total_	IF_Total/AREL_	IF_AR/AR_	IF_AREL/AR_	IF_AREL/AREL_	IF
Questionnaire score	Correlation coefficient	1.000	.698**	.692**	.715**	.713**	.692**	.687**
	Sig. (2-tailed)	.	.000	.000	.000	.000	.000	.000
IF_Total/Total_	Correlation coefficient	.698**	1.000	.999**	.965**	.956**	.999**	.935**
	Sig. (2-tailed)	.000	.	.000	.000	.000	.000	.000
IF_Total/AREL_	Correlation coefficient	.692**	.999**	1.000	.964**	.955**	1.000**	.934**
	Sig. (2-tailed)	.000	.000	.	.000	.000	.	.000
IF_AR/AR_	Correlation coefficient	.715**	.965**	.964**	1.000	.996**	.964**	.966**
	Sig. (2-tailed)	.000	.000	.000	.	.000	.000	.000
IF_AREL/AR_	Correlation coefficient	.713**	.956**	.955**	.996**	1.000	.955**	.967**
	Sig. (2-tailed)	.000	.000	.000	.000	.	.000	.000
IF_AREL/AREL_	Correlation coefficient	.692**	.999**	1.000**	.964**	.955**	1.000	.934**
	Sig. (2-tailed)	.000	.000	.	.000	.000	.	.000
IF	Correlation coefficient	.687**	.935**	.934**	.966**	.967**	.934**	1.000
	Sig. (2-tailed)	.000	.000	.000	.000	.000	.000	.

Note:

**means correlation is significant at the 0.01 level (2-tailed).

Table 3 shows that the five corrective IFs and traditional IF are significantly correlated with the questionnaire score for the 30 ophthalmologic journals. The correlation coefficients between traditional IF and the five corrective IFs are above 0.9. The traditional IF is most relevant to IF_AREL/AR_ and IF_AR/AR_, for which the correlation coefficients are 0.967 and 0.966, respectively. There are high correlations between the five corrective IFs. The correlation coefficients between IF_Total/Total_ and both IF_Total/AREL_ and IF_AREL/AREL_ reach 0.999. IF_AR/AR_ is the most relevant to IF_AREL/AR_, and IF_Total/AREL_ is most relevant to IF_AREL/AREL_. The questionnaire score is more relevant to the five corrective IFs than to traditional IF. IF_AR/AR_ is the most relevant to the questionnaire score, with a correlation coefficient of 0.715, followed by IF_AREL/AR_, for which the correlation coefficient is 0.713. The correlation coefficient between IF_Total/AREL_ and the questionnaire score is the same as that between IF_AREL/AREL_ and the questionnaire score, and the correlation coefficients for both are 0.692.

### Mathematical Journal Results

#### Quantity and citations for each type of document published by 27 mathematical journals

To determine the distribution of non-citable items in mathematics, we analyzed the quantity and number of citations for each type of document published by 27 mathematical journals. The results are shown in Table 4.

**Table 4:** Quantity and number of citations for each type of document published by 27 mathematical journals.

Journal title	Articles		Reviews		Editorialmaterials		Letters		Other items*	
	Amount	Citation	Amount	Citation	Amount	Citation	Amount	Citation	Amount	Citation
*Adv Math*	616	833	0	0	0	0	0	0	2	0
*Am J Math*	105	103	0	0	0	0	0	0	1	0
*Am Math Mon*	171	43	0	0	24	0	117	0	1	0
*P Am Math Soc*	843	575	0	0	1	0	0	0	3	0
*T Am Math Soc*	475	519	0	0	0	0	0	0	1	0
*Ann Math*	123	411	0	0	0	0	0	0	1	0
*Commun Pur Appl Math*	91	273	1	0	2	0	0	0	4	0
*Duke Math J*	135	223	0	0	0	0	0	0	5	0
*J Math Anal Appl*	1716	1901	0	0	3	2	0	0	14	2
*Indiana U Math J*	149	84	0	0	0	0	0	0	2	0
*Mich Math J*	86	35	0	0	1	0	0	0	0	0
*Pac J Math*	263	117	0	0	0	0	0	0	1	0
*Rocky Mt J Math*	208	81	0	0	0	0	0	0	0	0
*Mem Am Math Soc*	55	15	0	0	1	0	0	0	0	0
*Lect Notes Math*	498	101	0	0	86	6	0	0	2	0
*B Am Math Soc*	28	43	0	0	6	1	0	0	0	0
*Hist Math*	23	7	0	0	1	0	0	0	36	0
*Math Intell*	61	17	0	0	23	4	6	1	1	1
*Houston J Math*	158	61	0	0	0	0	0	0	0	0
*J Am Math Soc*	63	168	0	0	0	0	0	0	1	0
*Exp Math*	66	29	0	0	0	0	0	0	1	0
*Math Res Lett*	190	75	0	0	0	0	0	0	0	0
*New York J Math*	88	29	0	0	0	0	0	0	1	0
*Asian J Math*	62	30	0	0	0	0	0	0	1	0
*Pure Appl Math Q*	63	10	0	0	3	0	0	0	0	0
*Found Comput Math*	54	138	0	0	1	0	0	0	0	0
*Math Control Relat F*	41	24	0	0	1	0	0	0	0	0
*All Journals*	6431	5945	1	0	153	13	123	1	78	3

Note:

*means non-citable items such as biographical items, corrections and news items et al.

Table 4 shows that these mathematical journals mainly published articles, and only *Commun Pur Appl Math* published one review from 2012 to 2013. There were 153 editorials and 123 letters published by the mathematical journals, but only 78 other non-citable items. Compared with the number of articles, the number of non-citable items is very small. The citations to articles are far higher in number than those to non-citable items. The review published by *Commun Pur Appl Math* was not cited in 2014. The citations to the 153 editorials in 2014 numbered only 13, the 123 letters were just cited once in 2014, the citations to other non-citable items numbered only three.

#### Questionnaire score and various IFs of 27 mathematical journals

We analyzed 117 valid questionnaires to obtain the total expert score of 27 mathematical journals. In addition, we calculated the five corrective IF values for each journal and retrieved its traditional IF through the JCR database. The results are shown in Table 5.

**Table 5:** Questionnaire score and various IFs of 27 mathematical journals.

Journal title	Questionnaire score	IF_Total/Total_	IF_Total/AREL_	IF_AR/AR_	IF_AREL/AR_	IF_AREL/AREL_	IF
*Adv Math*	767.1	1.348	1.352	1.352	1.352	1.352	1.294
*Am J Math*	694.5	0.972	0.981	0.981	0.981	0.981	1.181
*Am Math Mon*	536.5	0.137	0.138	0.251	0.251	0.138	0.251
*P Am Math Soc*	708.8	0.679	0.681	0.682	0.682	0.681	0.681
*T Am Math Soc*	791.4	1.090	1.093	1.093	1.093	1.093	1.122
*Ann Math*	1023.1	3.315	3.341	3.341	3.341	3.341	3.236
*Commun Pur Appl Math*	549.2	2.786	2.904	2.967	2.967	2.904	3.13
*Duke Math J*	743.9	1.593	1.652	1.652	1.652	1.652	1.578
*J Math Anal Appl*	368.2	1.099	1.108	1.108	1.109	1.107	1.12
*Indiana U Math J*	433.5	0.556	0.564	0.564	0.564	0.564	0.577
*Mich Math J*	512.3	0.402	0.402	0.407	0.407	0.402	0.407
*Pac J Math*	579.8	0.443	0.445	0.445	0.445	0.445	0.433
*Rocky Mt J Math*	401.7	0.389	0.389	0.389	0.389	0.389	0.399
*Mem Am Math Soc*	671	0.268	0.268	0.273	0.273	0.268	1.727
*Lect Notes Math*	640.6	0.183	0.183	0.203	0.215	0.183	0.41
*B Am Math Soc*	733.7	1.294	1.294	1.536	1.571	1.294	2.107
*Hist Math*	142.1	0.117	0.292	0.304	0.304	0.292	0.435
*Math Intell*	292.9	0.253	0.256	0.279	0.361	0.244	0.295
*Houston J Math*	307.1	0.386	0.386	0.386	0.386	0.386	0.424
*J Am Math Soc*	879.9	2.625	2.667	2.667	2.667	2.667	2.556
*Exp Math*	292.7	0.433	0.439	0.439	0.439	0.439	0.424
*Math Res Lett*	408.5	0.395	0.395	0.395	0.395	0.395	0.411
*New York J Math*	279	0.326	0.330	0.330	0.330	0.330	0.33
*Asian J Math*	289	0.476	0.484	0.484	0.484	0.484	0.532
*Pure Appl Math Q*	211	0.152	0.152	0.159	0.159	0.152	0.175
*Found Comput Math*	160.5	2.509	2.509	2.556	2.556	2.509	2.389
*Math Control Relat F*	76.1	0.571	0.571	0.585	0.585	0.571	0.512

#### Correlations between questionnaire score and various IFs of 27 mathematical journals

**Fig 2:**
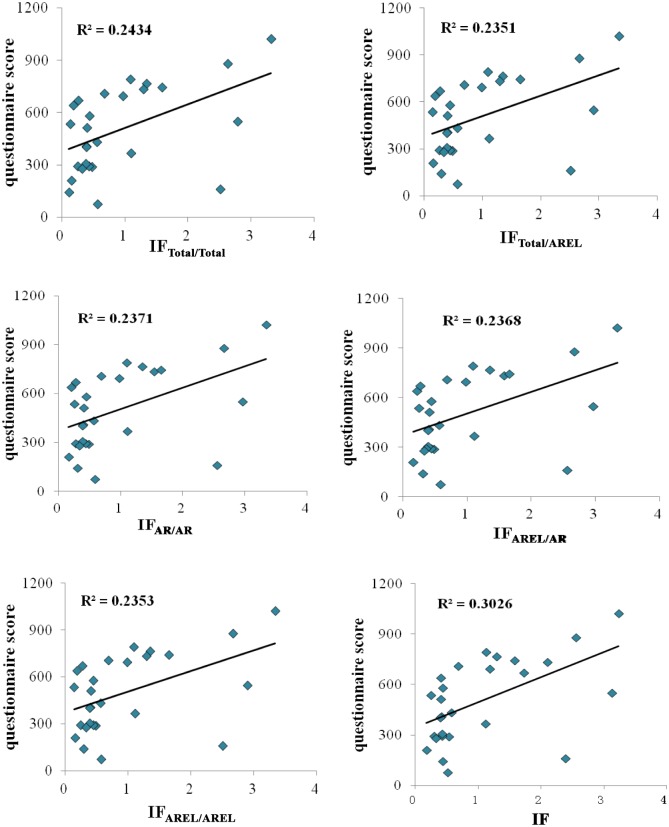
Correlations between the questionnaire score and various IFs of 27 mathematical journals.

Fig 2 shows the scatter diagrams of the relationships between the questionnaire score and various IFs of the mathematical journals. In addition, the K-S normal distribution test results show that the five corrective IFs and traditional IF are not subject to a normal distribution. Thus, we analyzed the relationships between the questionnaire score and corrective IFs using the Spearman rank correlation test. The results are shown in Table 6.

**Table 6:** Correlations between questionnaire score and various IFs of 27 mathematical journals.

		Questionnaire score	IF_Total/Total_	IF_Total/AREL_	IF_AR/AR_	IF_AREL/AR_	IF_AREL/AREL_	IF
Questionnaire score	Correlation coefficient	1.000	.510**	.477*	.474*	.480*	.477*	.545**
	Sig. (2-tailed)	.	.007	.012	.012	.011	.012	.003
IF_Total/Total_	Correlation coefficient	.510**	1.000	.991**	.988**	.989**	.991**	.849**
	Sig. (2-tailed)	.007	.	.000	.000	.000	.000	.000
IF_Total/AREL_	correlation coefficient	.477*	.991**	1.000	.997**	.994**	1.000**	.865**
	Sig. (2-tailed)	.012	.000	.	.000	.000	.	.000
IF_AR/AR_	Correlation coefficient	.474*	.988**	.997**	1.000	.998**	.997**	.852**
	Sig. (2-tailed)	.012	.000	.000	.	.000	.000	.000
IF_AREL/AR_	Correlation coefficient	.480*	.989**	.994**	.998**	1.000	.994**	.846**
	Sig. (2-tailed)	.011	.000	.000	.000	.	.000	.000
IF_AREL/AREL_	Correlation coefficient	.477*	.991**	1.000**	.997**	.994**	1.000	.865**
	Sig. (2-tailed)	.012	.000	.	.000	.000	.	.000
IF	Correlation coefficient	.545**	.849**	.865**	.852**	.846**	.865**	1.000
	Sig. (2-tailed)	.003	.000	.000	.000	.000	.000	.

Note:

* correlation is significant at the 0.01 level (2-tailed).

**correlation is significant at the 0.01 level (2-tailed).

Table 6 shows that the five corrective IFs and traditional IF are significantly correlated with questionnaire score for 27 mathematical journals. The correlation coefficients between the traditional IF and corrective IFs vary between 0.8 and 0.9. The traditional IF is most relevant to IF_Total/AREL_ and IF_AREL/AREL_, for which the correlation coefficients are both 0.865. Similar to ophthalmology, there are high correlations between the five corrective IFs. IF_Total/Total_ is most relevant to IF_Total/AREL_ and IF_AREL/AREL_, for which the correlation coefficients are both 0.991. IF_AR/AR_ is the most relevant to IF_AREL/AR_, and IF_AREL/AREL_ is most relevant to IF_AR/AR_. In contrast to ophthalmology, traditional IF is the most relevant to the questionnaire score, with a correlation coefficient of 0.545, followed by IF_Total/Total_, for which the correlation coefficient is 0.510. The correlation coefficient between IF_AR/AR_ and the questionnaire score is the lowest.

Above all, whether ophthalmologic journals or mathematical journals, corrective IFs are highly correlated with traditional IF, and there are high correlations between any pair of corrective IFs. The correlations between corrective IFs and the questionnaire score of the ophthalmologic journals are higher than those of the mathematical journals. For ophthalmologic journals, the effect of journal evaluation shows that the corrective IFs are superior to traditional IF; however, there is little value to using the corrective IFs for mathematical journals. The reason for this could be that ophthalmologic journals publish more non-citable items that attract more citations, while mathematical journals publish fewer non-citable items and these non-citable items have fewer citations. Therefore, the corrective effect of traditional IF might be related to the quantitative and citation characteristics of non-citable items published by journals in different disciplines.

## Conclusions

### Five Corrective IFs and traditional IF are highly correlated

For both ophthalmologic and mathematical journals, the five corrective IFs are significantly positively and highly correlated with traditional IF. Although both ophthalmologic journals and mathematical journals publish a certain number of non-citable items, their quantities and number of citations are very low compared with those of articles. The numerator and/or denominator in the traditional IF are correspondingly improved. Thus, although there are some differences in the quantity of source items used for the corrective IFs and traditional IF, these differences are not significant. For both ophthalmology and mathematics, the journal ranks based on the corrective IFs and traditional IF are largely consistent, especially for the mathematical journals.

In general, the correlations between the five corrective IFs and traditional IF for mathematical journals are lower than those for ophthalmologic journals. For ophthalmologic journals, the source items in the denominator for IF_AR/AR_ and IF_AREL/AR_, which correct the numerator of traditional IF, are the same as traditional IF. Among the non-citable items published by ophthalmologic journals, letters and editorials attract more citations; other non-citable items are rarely cited. Nevertheless, the number of citations to letters and editorials are much lower than those to articles and reviews. Hence, IF_AR/AR_ and IF_AREL/AR_ are highly correlated with traditional IF. The research results for mathematical journals are totally different from those of ophthalmologic journals. For mathematical journals, IF_Total/AREL_ and IF_AREL/AREL_ are the most correlated with traditional IF. IF_Total/AREL_ is constructed by re-defining the denominator in traditional IF, and IF_AREL/AREL_ is constructed by simultaneously correcting both numerator and denominator in the traditional IF. Therefore, because of the difference in subject nature, the correlations between corrective IFs and the traditional IF of journals are clearly different. The correlations between corrective IFs and traditional IF in other disciplines have yet to be studied.

### Five corrective IFs are highly correlated

The statistical analysis shows that there are high correlations between the five corrective IFs for both ophthalmologic and mathematical journals. For ophthalmologic journals, the only difference between IF_Total/Total_ and IF_Total/AREL_ is the denominator. Because document types published by ophthalmologic journals are relatively concentrated into articles, reviews, letters, and editorials, IF_Total/Total_ is highly correlated with IF_Total/AREL_. Articles, reviews, letters, and editorials have more citations than other non-citable items, so IF_AREL/AREL_ is highly correlated with IF_Total/Total_. In addition, citations to letters and editorials are far fewer than to articles and reviews, thus IF_AREL/AR_ is highly correlated with IF_AR/AR_. The research results for mathematical journals are the same as those for ophthalmologic journals.

### It is scientific and reasonable to use five corrective IFs for journal evaluation

The scatter plots show that there are significant linear correlations between the five corrective IFs and questionnaire score for both ophthalmologic and mathematical journals. Further statistical analysis shows that for both ophthalmologic journals and mathematical journals, the five corrective IFs are significantly correlated with the questionnaire score. For ophthalmologic journals, the correlation coefficients between the various corrective IFs and questionnaire score vary from 0.6 to 0.8. For mathematical journals, the correlation coefficients vary from 0.4 to 0.6. Therefore, for both ophthalmologic and mathematical journals, it is scientific and feasible to use all the corrective IFs for journal evaluation.

### Corrective effect of journal IF in different subjects is obviously different

The results of the study show that the correlations between five corrective IFs and questionnaire score for ophthalmologic journals are obviously higher than those for mathematical journals. For ophthalmologic journals, five corrective IFs are more relevant to the questionnaire score than to traditional IF. Therefore, the journal evaluation using the five corrective IFs is better than an evaluation using traditional IF. IF_AR/AR_ is the most relevant to the questionnaire score, followed by IF_AREL/AR_. Thus, we conclude that the corrective effect of IF_AR/AR_ is best for ophthalmologic journals, IF_AREL/AR_ is better than IF_Total/Total_, and the effects on journal evaluation from using IF_Total/AREL_ and IF_AREL/AREL_ are the worst. For mathematical journals, journal evaluation using the traditional IF is superior than that using corrective IFs. Among the five corrective IFs, the effect on journal evaluation of IF_Total/Total_ is the best, IF_AREL/AR_ is better than IF_Total/AREL_ and IF_AREL/AREL_, and the effect in journal evaluation of IF_AR/AR_ is the worst.

In conclusion, although the assumptions behind correcting traditional IF are reasonable in theory, our empirical study finds that not all subjects need to correct their journal IF. In our study, it is valuable to correct the traditional IF of ophthalmologic journals; however, it may not make much sense to correct the traditional IF of mathematical journals. Overall, the quantity and citations of non-citable items are lower than those of citable items. Hence, the corrective effect of IF might be not obvious for most disciplines, but it might be valuable to correct the traditional IF of journals publishing more editorials and letters.

## Supporting Information

S1 Dataset Data of questionnaire survey from American ophthalmologic researchers giving each American ophthalmology journal a score. (XLS)

S2 Dataset Data of questionnaire survey from American mathematical researchers giving each American mathematical journal a score. (XLS)

## References

[1] Garfield E. Citation Indexes for Science: a new dimension in documentation through association of ideas. Science. 1990; 122:108–111. 10.1126/science.122.3159.108 14385826

[2] Wu XF, Fu Q, Rousseau R. On indexing in the Web of Science and predicting journal impact factor. Journal of Zhejiang University SCIENCE B. 2008; 9:582–590. 10.1631/jzus.B0840001 18600790 PMC2443356

[3] Garfield E. The history and meaning of the journal impact factor. JAMA-Journal of the American Medical Association. 2006; 295:90–93. 10.1001/jama.295.1.90 16391221

[4] Wan H, Tan ZY, Lu JJ, Zhu XL. Summary of the Evolution of Citation Analysis Research: 2001–2014. Library and Information Service. 2015; 59:120–136.

[5] Garfield E, Sher IH. New factors in the evaluation of scientific literature through citation indexing. American Documentation. 1963; 14(3):195–201.

[6] Garfield E. Citation analysis as a tool in journal evaluation. Science & Justice. 1972; 178:471–479. 10.1126/science.178.4060.471 5079701

[7] Campanario JM. Large increases and decreases in journal impact factors in only one year: The effect of journal self-citations. Journal of the American Society for Information Science and Technology. 2011; 62:230–235.

[8] Fersht A. The most influential journals: Impact Factor and Eigenfactor. Proceedings of the National Academy of Sciences of the United States of America. 2009; 106: 6883–6884. 10.1073/pnas.0903307106 19380731 PMC2678438

[9] Liu XL. Structural characteristics of impact factors of the ten top international journals. Acta Editologica. 2014; 26:296–300.

[10] Gai SS, Liu XL, Zhang SL. Methodology of calculation and structural analysis of journal impact factor based on Web of Science database: a case study of *Nature*. Chinese Journal of Scientific and Technical Periodicals. 2014; 25:980–984.

[11] Journal Citation Reports. Journal Source Data[EB/OL]. (2015-10-28) [2012-05-22]. http://admin-apps.webofknowledge.com/JCR/help/h_sourcedata.htm#sourcedata.

[12] Moed HF, Vanleeuwen TN. Improving the accuracy of Institute for Scientific Informations journal impact factors. Journal of the Association for Information Science and Technology. 1995; 46:461–467.

[13] Liu XL, Gai SS, Zhang SL, Zhou J. Citation characteristics of non-citable documents and their contribution to journal impact factor. Acta Editologica. 2015; 27:495–499.

[14] Opthof T. Impact factor 2013 of the Netherlands Heart Journal Surpasses 2.0. Netherlands Heart Journal. 2014; 22: 167–138. 24574316 10.1007/s12471-014-0538-8 PMC3954925

[15] Van Leeuwen T. Discussing some basic critique on journal impact factors: revision of earlier comments. Scientometrics. 2012; 92: 443–455. 22844166 10.1007/s11192-012-0677-x PMC3399074

[16] Bornmann L, Neuhaus C, Daniel HD. The effect of a two-stage publication process on the journal impact factor: a case study on the interactive open access journal Atmospheric Chemistry and Physics. Scientometrics. 2011; 86:93–97.

[17] McKerahan TL, Carmichael SW. What is the impact factor, anyway?. Clinical Anatomy. 2012; 25:283–283. 10.1002/ca.21291 22034097

[18] Bensman SJ. Distributional differences of the impact factor in the sciences versus the social sciences: an analysis of the probabilistic structure of the 2005 journal citation reports. Journal of the American Society for Information Science and Technology. 2008; 59: 1366–1382.

[19] Campanario JM, Gonzalez L. Journal self-citations that contribute to the impact factor: Documents labeled "editorial material" in journals covered by the Science Citation Index. Scientometrics. 2006; 69: 365–386.

[20] Gonzalez L, Campanario JM. Structure of the impact factor of journals included in the Social Sciences Citation Index: Citations from documents labeled "editorial material". Journal of the American Society for Information Science and Technology. 2007; 58: 252–262.

[21] Jacso P. Five-year impact factor data in the Journal Citation Reports. Online Information Review. 2009; 33: 603–614.

[22] Jones AW. The distribution of forensic journals, reflections on authorship practices, peer-review and role of the impact factor. Forensic Science International. 2007; 165:115–128. 16784827 10.1016/j.forsciint.2006.05.013

[23] Fleck C. The impact factor fetishism. Arch Eur Sociol. 2013; 54: 327–356.

[24] Jackson A. The impact factor game: the rising impact factor of the British Journal of Radiology—a success story? British Journal of Radiology. 2010; 83: 93–98. 10.1259/bjr/18689409 20139258 PMC3473528

[25] Reynolds JC, Menegazzi JJ, Yealy DM. Emergency medicine journal impact factor and change compared to other medical and surgical specialties. Academic Emergency Medicine. 2012; 19: 1248–1254. 10.1111/acem.12017 23167855

[26] Wang P, Liu XL, Liu RY, Zheng CM. Review on SNIP and Revised SNIP2 Based on Scopus Database. Chinese Journal of Scientific and Technical Periodicals. 2013; 24(5):838–842.

[27] Gonzalez-Pereira B, Guerrero-Bote VP, Moya-Anegon F. A new approach to the metric of journals' scientific prestige: The SJR indicator. Journal of Informetrics. 2010; 4(3): 379–391.

[28] Xu HY, Fang S. Study on impact factor based on ordering relations converted to weight factor according to the types of journal literature. Journal of Intelligence. 2012; 31:50–53.

[29] Tort ABL, Targino ZH, Amaral OB. Rising publication delays inflate journal impact factors. PLOS ONE. 2012; 7: 1–6. 10.1371/journal.pone.0053374 PMC3534064 23300920

[30] Wu YS. The definition of impact factor has better be adjusted. [EB/OL]. (2014-06-25) [2015-09-25]. Available: http://blog.sciencenet.cn/blog-1557-806325.html.

[31] Herna´n MA. Epidemiologists (of all people) should question journal impact factors (with discussion). Epidemiology. 2008; 19: 366–368. 18414080 10.1097/EDE.0b013e31816a9e28

[32] Pendlebury DA, Adams J. Comments on a critique of the Thomson Reuters journal impact factor. Scientometrics. 2012; 92: 395–401.

[33] Moed HF, vanLeeuwen TN, Reedijk J. A critical analysis of the journal impact factors of Angewandte Chemie and the Journal of the American Chemical Society—Inaccuracies in published impact factors based on overall citations only. Scientometrics. 1996; 37(1): 105–116.

[34] Lozano GA, Lariviere V, Gingras Y. The weakening relationship between the impact factor and papers’ citations in the digital age. Journal of the American Society for Information Science and Technology. 2012; 63: 2140–2145.

[35] Dorta-Gonzalez P, Dorta-Gonzalez MI, Santos-Penate DR, Suarez-Vega R. Journal topic citation potential and between-field comparisons: the topic normalized impact factor. Journal of Informetrics. 2014; 8: 406–418.

[36] Brzezinski M. Empirical modeling of the impact factor distribution. Journal of Informetrics. 2014; 8: 362–368.

[37] Van Nierop E. The introduction of the 5-year impact factor: does it benefit statistics journals?. Statistica Neerlandica. 2010; 64: 71–76.

[38] Harnad S. Validating Research Performance Metrics against Peer Rankings. Ethics in Science and Environmental Politic. 2008; 11: 103–107.

[39] Weale AR, Bailey M, Lear P. The Level of Non-Citation of Articles within a Journal as a Measure of Quality: a Comparison to the Impact Factor. BMC Medical Research Methodology. 2004; 4: 1–8. 10.1186/1471-2288-4-14 PMC434502 15169549

